# Collaboration between an NHS University Teaching Hospital and independent hospital to maintain CT colonography service provision during the 2020 COVID-19 pandemic

**DOI:** 10.1259/bjro.20210025

**Published:** 2022-06-03

**Authors:** Paul Holland, Deborah De Abreu, Yutaro Higashi, Christopher GD Clarke

**Affiliations:** Department of Clinical Radiology, Nottingham University Hospitals NHS Trust, Derby Road, Nottingham NG7 2UH, UK

## Abstract

Our trust performed CTCs at 93% of the capacity of the previous year, scanning 1265 patients in 2020, compared with 1348 in 2019. We describe the changes made to our service to achieve this, which included collaboration with the colorectal surgical team to prioritise existing CTC patients according to faecal-immunochemical tests and full blood count results, and the associated challenges which included image transfer delays and patient attendance for scans. Furthermore, the endoscopy and radiology services used the opportunity created by co-location at the same hospital site to provide a same day incomplete colonoscopy and staging service for optically confirmed cancers.

Collaboration between the NHS and independent sector allowed us to achieve continuity of service provision during the height of the COVID-19 pandemic without substituting unprepared CT abdomen and pelvis instead of the more sensitive CTC.

## Introduction

Colorectal cancer (CRC) is the third most common cancer in the UK and the second commonest cause of cancer death in the UK.^1^ It is estimated that the 2-week wait (2WW) colorectal referral pathway diagnoses 33–34% of all colorectal cancer in England and Wales per year.^2^ Optical colonoscopy remains the reference standard investigation for CRC, however CT colonography (CTC) can also be used to provide a similarly sensitive alternative to colonoscopy for detecting cancer and large polyps.^3^

Nottingham University Hospitals NHS Trust (NUH) is one of the largest teaching hospitals in England with 1700 beds.^4^ Prior to the COVID-19 pandemic, referrals for CTC examinations at our trust were increasing steadily with 1348 CTC’s performed in 2019 and our second busiest month on record was February 2020 with 143 CTCs performed. It was predicted that this trend of increased referrals would continue into 2020 and provisions had been made to continue with this level of capacity to meet the referral numbers. All CTC 2WW urgent suspected colorectal cancer referrals required a faecal-immunochemical test (FIT), a non-invasive investigation for detecting the presence of blood within the stool. A recent service evaluation at NUH found that when used alongside blood tests for anaemia, a FIT result of ≥4 µg Hb/g (with the exception of patients with rectal bleeding) had a sensitivity of 99.8% and specificity of 61.4% for colorectal cancer diagnosis.^5^ We also accepted some CTC referrals outside of the 2WW pathway based on clinical symptoms and other risk factors alone if not suitable for optical colonoscopy (*e.g.* from gastroenterology).

We describe why and how we changed our CTC pathway from March to September of 2020 in response to the challenges of the pandemic, and the effect on our CTC service.

### Impact on the CTC service due to COVID-19 in March 2020

In March 2020 in response to the coronavirus (COVID-19) pandemic in the UK, NHS services were temporarily suspended or scaled back in order to maintain emergency care and potentially reduce infection rates. Between April and June 2020, it has been estimated that there were 1.32–1.5 million fewer elective admissions than would normally be expected and furthermore around 250,00 fewer urgent cancer referrals.^6^ On 23 March 2020, the British Society of Gastroenterology (BSG) and Joint Advisory Group on GI endoscopy (JAG) updated their guidance on the use of endoscopy during the COVID-19 pandemic and recommended that all non-emergency endoscopy stopped immediately.^7^ This included most patients undergoing optical colonoscopy for CRC diagnoses. Following this statement, our local endoscopy services halted non-emergency work leading to an immediate increased demand for CTC.

2 days later, on 25 March 2020, The British Society of Gastrointestinal and Abdominal Radiology (BSGAR) recommended that CTC should also stop unless there was explicit local agreement amongst all relevant stakeholders that capacity exists to continue a reduced service.^8^ The reasoning behind this decision was multifactorial. The population referred for CTC had a high proportion of older and/or frail patients, often with comorbidities such as cardiovascular and respiratory disease.^8^ In March 2020, the COVID-19 mortality rate was thought to be around 5% in those aged 70–79 and over 9% in those aged 80+, which compares to the relatively low incidence of a CRC diagnosis in those referred via a 2WW pathway (3–7%) and the incidence on CTC is in the lower part of this range.^8^ There was also concern at the time that COVID-19 may be excreted in faeces and this was considered as a risk for the transmission of COVID-19. Furthermore, although lower GI endoscopy was not considered an aerosol generating procedure (AGP), this was under review at the time and the true transmission risk was unknown.

In addition, the introduction of safeguarding measures for patients and staff included a requirement to wear additional personal protective equipment (PPE) including a surgical mask, apron, and gloves, and thoroughly clean the scanner between patients to reduce the risk of spreading infection. These changes meant that each CTC examination took longer than usual to perform. With the rapidly increasing prevalence of COVID-19 cases within the inpatient population, it was necessary to designate physical scanner space within the hospital as COVID-19 and non-COVID-19 areas. This required both patient transfer pathway alterations as well as infrastructural modifications to corridors and waiting areas. At Nottingham University Hospitals(NUH), we required a designated COVID-19 CT scanner at each of our two hospital sites to meet the acute work demand. Isolating an entire scanner (and waiting area) for this “covid” work resulted in a predictable decrease in non-COVID-19 capacity, particularly in reference to elective outpatient work and vastly reduced the capacity to perform ‘clean’ outpatient CT examinations.

This combination of factors led to a vastly reduced capacity to undertake CTC along with increased numbers of patients referred for CTC (due to halting of endoscopy services), therefore we could not meet the demand for CTC and had to make major changes to the service.

### Changes to the service in response to COVID-19

On 25 March, 2020 our CTC service was temporarily halted in response to the pandemic. As a consequence of this, alongside the trust’s acute COVID-19 response there was a backlog of requests for CTC for patients requiring urgent lower GI tract investigation that could not be addressed. In order to prioritise the patients referred for CTC, an urgent meeting was arranged between the local colorectal surgical service and radiology. We decided that rather than choosing a suboptimal examination such as an unprepared CT abdomen and pelvis, with a view to future colonoscopy when deemed safe, we should make efforts in delivering the correct examination for the patient at the first time of asking. In this instance, within the confines of the national guidance, this examination was considered to be a CTC. To facilitate this, there was a need to evaluate and manage the combination of increased demand with reduced capacity.

A revised pathway was therefore developed using a combination of FIT, ferritin, platelets, haemoglobin (Hb) and clinical symptoms to aid prioritisation of each patient. Our criteria were based on then local unpublished data analysing a cohort of 13,361 patients on 2WW colorectal cancer referral pathway.^9^ In those patients with a FIT >100 µg Hb/g, the CRC detection rate was 20.7%.^9^ The CRC detection rate in patients with FIT 10–19.9 µg Hb/g was 1.4% and the overall CRC rate in patients with FIT <20 µg Hb/g was less than 0.3% during the follow-up period, both of which are well below the recommended 3% threshold for NICE urgent cancer referrals.^9,10^

This new pathway agreed between radiology and the colorectal service was used by radiologists and radiographers to justify scans and prioritise the referrals based on the probability of detecting significant polyps/cancer, thus avoiding imaging patients deemed low risk for colorectal cancer and ensuring our highest risk patients were not delayed in the backlog that had accumulated. Patient bookings for CTC were prioritised using these results (Table 1) with ASAP patients appointed first until that backlog was cleared followed by priority 1–3 patients in ascending order. New referrals were assigned a priority when authorised by radiology.

**Table 1.** Criteria for prioritisation of CTC requests for 2WW colorectal cancer referrals during the COVID-19 recovery period

FIT result		Blood results of: Ferritin <25 or>350 ug ml^−1^ or Haemoglobin <130 (male) or <120 (female) g/L or Platelets > 400×10^9^ l^−1^	Priority
>100–149.9 µg Hb/g		Ignore	ASAP
>20–99.9 µg Hb/g	&	Abnormal	1
>4–20 µg Hb/g		Abnormal	2
>20–99.9 µg Hb/g		Normal	3
>4–20 µg Hb/g		Normal	Discharge back to referrer

CTC, CT colonography; FIT, faecal-immunochemical test; 2WW, 2-week wait.

Radiologists then manually rejustified all the outstanding CTCs based on the FIT level, prioritising them according to Table 1. When rejustifying, we also had a lower threshold for rediscussing cases with the referring clinician if we suspected the patients were not surgical candidates to ensure priority went to those patients most likely to benefit from the investigation.

To overcome the local issue of scanner capacity, on 7 April 2020, we started undertaking CTC at a nearby independent provider (Spire Nottingham Hospital). Initially capacity at the independent hospital was two morning sessions per week staffed by a team of three advanced practice CTC radiographers. This allowed for one radiographer to operate the scanner and review the images, while the two carried out the procedure in the scan room. As many of the CTC patients had limited mobility, it was felt two members of staff were needed in the scanning room for safety. The team shared the responsibilities of consenting, checking on patients post-procedure and arranging patients referred from endoscopy. The CT equipment had an identical user interface to one already in use at our main NHS hospital site and the environment at the independent provider was ‘covid safe’ with no active covid patient admissions on site.

Our CTC imaging protocol consisted of a standard bowel prep regime of four doses of 50 ml Gastrografin over the 2 days preceding the CTC, taken in the morning and evening of each day (total 200 ml). This is in addition to a low residue diet with the last solid meal at lunch time the day before the CTC appointment. This prep regime remained consistent throughout 2019 and 2020.

To facilitate this move to the independent hospital, a local agreement was arranged between the NHS and provider to cover staff indemnity. The information and communications technology (ICT) systems were not connected, so a mobile workstation with a virtual private network (VPN) was installed to enable the team to access the radiology information system (RIS). Acquired images were reviewed on site by the radiographers on the local picture archive and communication system (PACS). These were then transferred to the NUH PACS system via an Image Exchange Portal (IEP). Experienced radiologists interpreted the CTC scans remotely using teleradiology, either working from home or from their main NHS hospital site. The effectiveness of teleradiology for CTC reporting by expert readers has previously been confirmed in large European trials on screening CTC.^11,12^

Radiographers were working away from the main NHS hospital and there was not a radiologist on site, therefore Standard Operating Procedures (SOPs) were developed to ensure safe delivery of this service. These covered radiographer training in vetting/protocolling examinations, review of colonic imaging to determine whether additional colonic views, CT chest or intravenous contrast was required. It is routine practice for NUH colonography practitioners to review the colonic components of the examination. There was close liaison between the radiologists and radiographers to allow for contact if there was an issue with the patient during the procedure such as perforation or contrast allergy. Gastrointestinal radiologists made themselves available via phone and MS Teams (Microsoft) as it was impractical for them to be at the independent hospital site as they were also required to support acute services at the NHS trust. Capacity was increased as systems of work were improved, and the NHS staff became more confident in their new working environment. Members of the colorectal surgical team were also deployed at the independent provider; therefore, medical cover was available in case there were complications requiring urgent medical review.

### Discussion and impact on our service

As a consequence of temporarily halting our CTC service, and the impact of new COVID-19 measures put in place by our trust, there was a growing backlog of patients requiring urgent lower GI investigation. Through utilising capacity within the independent sector, by July 2020, capacity was back to the level of service provided in the same period in 2019. This was further aided by running additional limited CTC lists in one of the CT scanners on site at NUH due to a scanner being stepped down from designated COVID-19 only, as our acute inpatient volume of COVID-19-related imaging reduced following the first UK national lockdown. This new capacity at NUH meant that CTC lists occasionally ran with only two CTC radiographers (previously three), but staff had gained enough confidence through their experience at remote site working to reduce staffing numbers.

**Figure 1.**
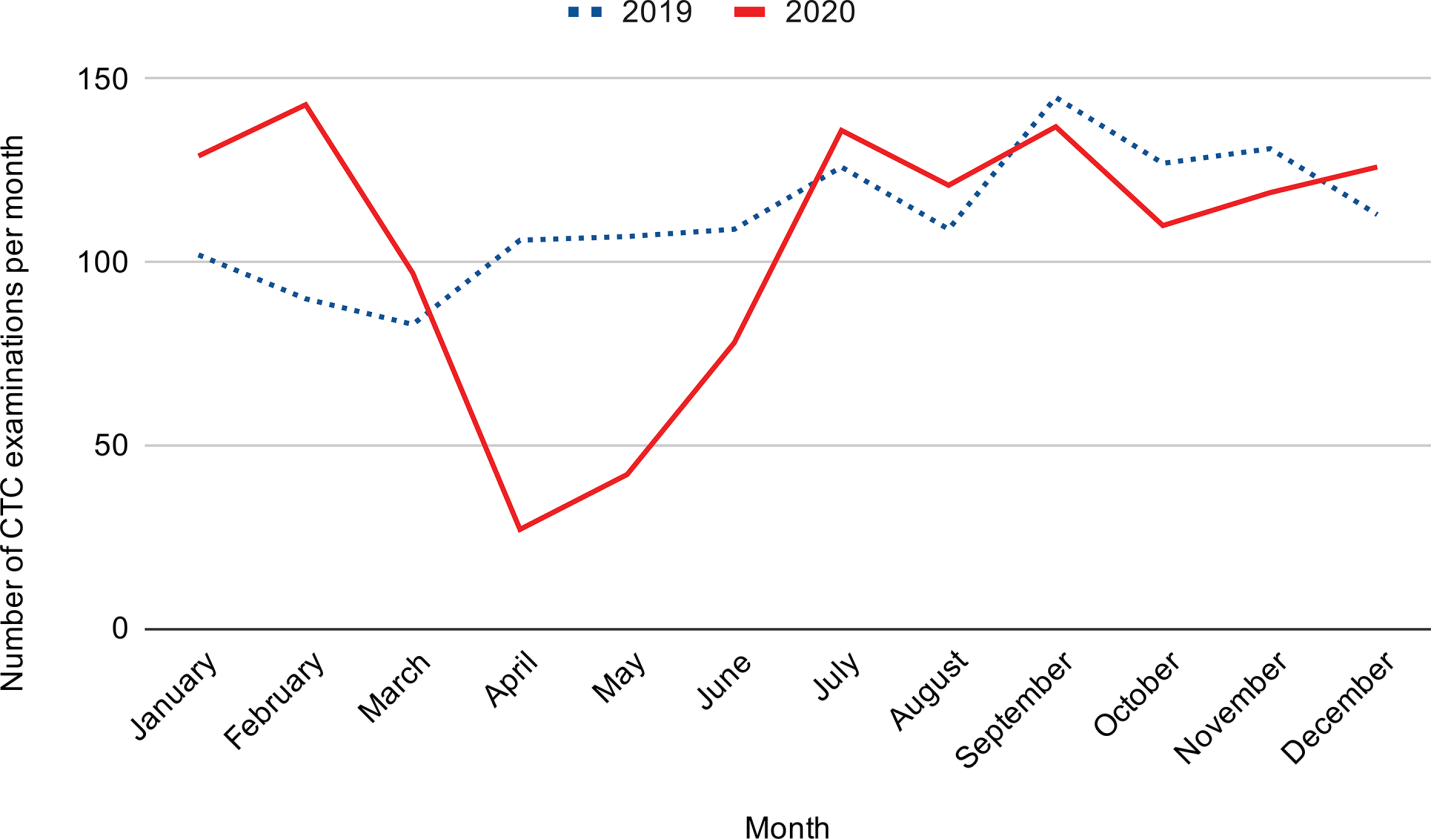
Number of CTC examinations performed at NUHs NHS Trust and on NHS patients in the independent sector in 2019 *vs* 2020. CT colonography; NUH, Nottingham University Hospitals.

Overall, in 2020, NUH performed CTCs at 93.8% of the capacity of the previous year with 1265 examinations completed with the help of the independent hospital compared with 1348 in 2019 (Table 2/Figure 1). The number of CTC referrals cancelled during 2020 (1 January 2020–1 December 2020) was 111 (8.1%), compared to 95 patients (6.6%) during the same period in 2019.

**Table 2.** Number of CTC examinations performed each month in 2019 and 2020 at Nottingham University Hospitals NHS Trust and Spire Nottingham Hospital

	Jan	Feb	March	April	May	June	July	Aug	Sept	Oct	Nov	Dec	Total
Nottingham University Hospitals 2019	102	90	83	106	107	109	126	109	145	127	131	113	1348
Nottingham University Hospitals 2020	129	143	97	0	0	8	33	26	34	19	23	126	1265
Spire Nottingham Hospital 2020^a^	0	0	0	27	42	70	103	95	103	91	96	0	

CTC, CT colonography.

The relocation of the CTC service to the independent hospital was not without its challenges.

a Nottingham University Hospital CTC scans were performed at Spire Nottingham Hospital in 2019.

Access to the images acquired at the independent centre at NUH was not instant as they needed to be transferred via image exchange portal (IEP) then merged into the correct folders on PACS by our PACS team, this slowed down report verifying times as this added 1–2 days to the process. If colorectal lesions or any new primary cancer were discovered at the time of scanning, a high priority was put on the IEP transfer to enable faster report verification. Whilst redeployed at the independent site, not every CTC radiographer was familiar with using the CT scanner there, so support was given from fellow CTC radiographer colleagues and radiographers at the site to overcome any training issues. As radiologists were not on-site and images were not available instantly on PACS, there were limitations to the support available clinically for the CTC radiographers as the radiologists could not see the images if the radiographers had queries. To mitigate this, all of the CTC radiographers in the NUH team hold a post-graduate certificate in CTC or were currently enrolled on the course and working towards one. Furthermore, there was a high level of experience amongst the radiographers, ranging from 2 to 5 years since completing their post-graduate certificate in CTC, and they were comfortable working autonomously, aided by several SOPs in place to allow the radiographers to add a CT chest or intravenous contrast when intracolonic pathology was seen, for example. The radiographer’s reporting experience of the colonic elements of the examination also gave the GI radiologists confidence that if there was significant pathology it would likely be spotted and escalated accordingly.

Patients were sometimes reluctant in attending for their appointment even though the independent site was relatively COVID secure compared to the other hospital sites which were seeing COVID-19 admission numbers rise.

Prior to the pandemic patients were asked to attend the radiology department to collect the Gastrografin bowel preparation. The reasoning for this was twofold; as Gastrografin is stored in glass bottles it is not safe to post and seeing the patient in person allows CT staff to establish that the patient understands the procedure they have been referred for and that they will tolerate it well. During the pandemic, to reduce footfall at NUH the CTC radiographers would phone the more vulnerable patients to have these discussions and the Gastrografin was then sent via taxi to them. Taxis were also used to transport patients with no alternative means of transport to the Spire Nottingham Hospital due to its remote location and the perceived risk and government’s advice to avoid public transport.^13^ The total spent on taxis between April and December 2020 under the “COVID expenditure code” was £1988.42. This was not a service offered previously and so no comparison can be made. The CTC service was the main user of this code, however this total cost may also include other COVID-19 related uses of taxi services in radiology. These measures all worked to keep our non-attendance rates low.

During the initial early stages of the pandemic and the AGP concern associated with CTC, the colonography radiographers were using full length gowns and FFP3 masks for every case.^8^ Due to the nature of the examination and the level of communication required whilst carrying out the procedure, the wearing of this mask made verbal communication with the patient challenging, furthermore the CTC radiographer was confined to the scanning room and adjacent corridor making dialogue with colleagues prohibitive. Appointment times were increased to 40 min slots per patient to allow for these additional requirements and for thorough cleaning after each patient. These longer slots also ensured good social distancing in the waiting room.

Other services within the trust continued to use radiology throughout the pandemic and so a balance had to be found which allowed continuation of CTC’s while also maintaining acute services. The CTC team in the trust is comprised of seven GI consultant radiologists and seven advanced practice radiographers, all of whom had responsibilities outside the CTC service. Rotas to ensure cover were made and flexibility was required of all members of the team to cover sick or self-isolation leave. As restrictions were eased and services that had previously been put on hold resumed this became increasingly difficult, but the CTC service was preserved.

The reorganisation of our service also created some opportunities to improve the service. The endoscopy service also moved to the independent hospital during the pandemic which allowed the opportunity to create a one stop surgical assessment, colonoscopy, and radiological staging pathway for patients, so that a patient with incomplete optical colonoscopy could have a completion CTC the same day. We also created a pathway for patients with a confirmed malignancy on colonoscopy to have their staging CT on the same day. A vetting protocol was created for the CTC radiographers who were then able to justify/authorise and scan these patients making the patient pathway more efficient. Over the period between April and September 2020, 18 patients had a CTC on the same day as their colonoscopy and 39 patients had a same day CT of the chest, abdomen and pelvis for staging of a colorectal cancer found on endoscopy. In total, 57 patients had their pathway shortened and did not need to reattend the hospital at another date. This pathway was previously trialled at NUH but had been less successful due to the pressures of balancing elective but unplanned work amongst the acute demands within the trust. The advantage of an elective only service at the independent provider was the key to the success in this instance. This same day service reduced footfall in the hospitals and streamlined the patient pathways.

## Conclusion

Funding made available by NHS England enabled collaboration with a local independent healthcare provider to allow continuation of the existing CTC service during the initial COVID-19 pandemic and clinically high-risk patients for colorectal cancer were prioritised for CTC and scheduled accordingly. The most suitable examination for the patient was delivered by radiology as per the BSGAR guidelines and no CTC referrals were changed to a standard CT abdomen and pelvis due to lack of capacity. A pathway for same day CTC following incomplete colonoscopy was also adopted on site, this proved successful and beneficial to the patients and 18 of these cases were performed between April and July 2020.
